# Impedimetric Biosensors for the Quantification of Serum Biomarkers for Early Detection of Lung Cancer

**DOI:** 10.3390/bios14120624

**Published:** 2024-12-18

**Authors:** Mahdi Arabnejad, Ibtisam E. Tothill, Iva Chianella

**Affiliations:** 1 Faculty of Engineering and Applied Sciences, Cranfield University, Cranfield, Bedfordshire MK43 0AL, UK; mahdia@silveray.co.uk; 2 Silveray, Stockport Road West, Stockport SK6 2BP, UK

**Keywords:** electrochemical impedance spectroscopy, neuron-specific enolase, carcinoembryonic antigen, immunosensors, magnetic nanobeads

## Abstract

Lung cancer is the most common type of cancer diagnosed worldwide and is also among the most fatal. Early detection, before symptoms become evident, is fundamental for patients’ survival. Therefore, several lung cancer biomarkers have been proposed to enable a prompt diagnosis, including neuron-specific enolase (NSE) and carcinoembryonic antigen (CEA). NSE and CEA are two serum proteins whose elevated levels have been associated with lung cancer. Hence, in this study, impedimetric biosensors (immunosensors) able to quantify NSE and CEA were developed as proof-of-concept devices for lung cancer diagnosis. The sensing platform exploited for the immunosensors comprises a novel combination of a magnetic platform, screen-printed gold electrode (SPGE), and magnetic nanobeads (MB). The MB were functionalized with antibodies to capture the analyte from the sample and to move it over the sensing area. The immunosensors were then developed by immobilizing another set of antibodies for either CEA or NSE on the SPGE through formation of self-assembled monolayer (SAM). The second set of antibodies enabled a sandwich assay to be formed on the surface of the sensor, while MB manipulation was applied during the sensor performance to depict a microfluidic system and increase antigen–antibody complex formation prior to CEA or NSE detection and quantification. The optimized immunosensors were successfully tested to measure various concentrations of CEA and NSE (0–100 ng/mL) in both phosphate buffer and 100% human serum samples. Clinically relevant detection limits of 0.26 ng/mL and 0.18 ng/mL in buffer and 0.76 ng/mL and 0.52 ng/mL in 100% serum for CEA and NSE, respectively, were achieved via electrochemical impedance spectroscopy with the use of potassium ferri/ferrocyanide as a redox probe. Hence, the two immunosensors demonstrated great potential as tools to be implemented for the early detection of lung cancer.

## 1. Introduction

Cancer is a major public health and economic problem, responsible for almost one in six deaths (16.8%) worldwide [1]. In 2022, 19.3 million cancer cases were diagnosed globally, and 10 million deaths were caused by cancer [1]. Among the different types of cancers, lung cancer was the most diagnosed (2.5 million new cases, 12.5% of all cancers worldwide) and was also the leading cause of cancer death, with an estimated 1.8 million deaths (18.7%) [1]. There are several types of lung cancers, and these are divided into two sub-groups: non-small cell lung cancer (NSCLC) and small cell lung cancer (SCLC). Whereas around 15–20% of lung cancers are SCLC and are usually associated with smoking, NSCLC is responsible for 80% to 90% of cases [2,3] and there is not a direct association with smoking. The most effective treatment method for lung cancer is surgery; however, most patients (75%) cannot be treated by surgery due to late diagnosis, which reduces survival rate and increases the chance of cancer recurring [4]. Thus, it is crucial to diagnose lung cancer at its early stages to increase survival. Cancer biomarkers are valuable, as measuring their concentration within body fluids, e.g.**,** serum, can be used for early detection. In fact, their serum levels can become abnormal at an early stage of the disease, well before patients experience physical symptoms and before the cancer has spread. Serum levels of biomarkers can also change as the cancer is treated; hence their monitoring can advise on doses and on the treatment’s efficacy, enabling precision medicine with improved patients’ clinical outcomes [5]. Detection and quantification of cancer biomarkers are expensive, time-consuming, and require highly trained personnel [6] and thus need to be conducted in specialized laboratory settings. In contrast, point-of-care testing (POCT) is recognized as a valuable tool to speed diagnosis, optimize patients care and reduce costs [7]. Biosensors, which are low-cost devices and non- (or minimally-) invasive analytical tools, exploit the ability of a sensing element (e.g., antibody, enzyme, DNA/RNA sequences) to recognize specifically an analyte of interest (e.g., a biomarker). Such sensing elements are intimately integrated with a physicochemical transducer (e.g., electrodes, optical or gravimetric chips), enabling the transformation of the recognition reaction into a measurable signal (e.g., resistance, current, optical, or acoustic signals) [8]. Biosensors are suitable for POCT [9] and can be used for quantifying biomarkers’ concentration to enable an early diagnosis of cancer and to monitor therapy [5].

Biomarkers can be found in tissues, breath, and other body fluids (e.g., serum, saliva, urine, etc.). Among the sources of lung cancer biomarkers, blood/serum can be considered one of the best choices. Easy access, the ability to continuously monitor the disease, and the release in serum of biomarkers at a very early stage of the cancer are the advantages of using serum samples for cancer biomarker detection and quantification [10,11,12]. Several serum biomarkers have been identified and reported in the literature, with both carcinoembryonic antigen (CEA) and neuron-specific enolase (NSE) among the most promising ones.

CEA is a human glycoprotein involved in cell adhesion. CEA has a molecular weight of 180 kD and is usually produced during fetal development. The glycoprotein is normally present at very low levels (<3 ng/mL) in healthy individuals. Although its level is slightly raised in people who smoke cigarettes (~5 ng/mL), its concentration increases significantly in patients with lung cancer [11,13,14,15,16]. CEA is known as one of the most specific and reliable biomarkers with a cut-off value around 5–7 ng/mL. It can be used for diagnosis not only of lung cancer but also breast, colorectal, ovarian, and colon cancers. The concentration level of CEA arises in serum after cancer incidence and during its progression, reaching levels higher than 100 ng/mL. Thereby, detection and quantification of CEA levels can enable an early cancer diagnosis as well as the monitoring of treatment [17,18,19]. In recent years, numerous studies have been carried out to develop electrochemical [20,21,22,23], optical [24,25,26] and magnetic [27] biosensors for the detection of CEA, with many utilizing nanomaterials and other novel materials such as metal–organic frameworks (MOFs) to enhance sensitivity and specificity [11,28,29,30].

Enolase is a 78 kDa glycolytic enzyme that consists of αα, ββ, γγ, αγ, and βγ subunits known as isozymes [31]. Since the isozyme with the γ subunit is found in neurons and endocrine cells, it is also known as neuron-specific enolase (NSE) [11,32,33,34]. NSE is currently one of the most reliable tumor markers in the diagnosis, prognosis, and follow-up of SCLC, even though increased levels of NSE have been reported also in NSCLC [35,36]. The level of NSE correlates with tumor burden, number of metastatic sites, and response to treatment [37]. In addition, NSE has been recognized to be a reliable, sensitive, and specific biomarker associated with neuroendocrine cancer and neuroblastoma [11,32,33,34,38]. Although strokes can also cause elevation of NSE concentration in blood/serum, the concentration returns to normal levels within a week from the event [39]. NSE serum concentration range in healthy individuals is between 5 and 12 ng/mL, and it rises significantly in the presence of cancer. Particularly high levels of NSE (>100 ng/mL) have been found in patients with SCLC, and the concentration can increase to nearly 1 µg/mL in patients with late-stage cancer [38,40,41]. Hence, the concentration of NSE in serum can be used as an indicator for both early cancer diagnosis and response of patients to treatment. In recent years a few studies have been reported on biosensors for NSE detection in serum using a variety of sensing techniques such as electrochemical impedance spectroscopy (EIS) [42,43], quantum dots [41,44,45], differential pulse voltammetry [13,46,47], voltammetry [48], optical methods [49,50,51] and amperometry [38].

EIS is a powerful electrochemical technique that is used to estimate the electrical resistance of a system (or of an equivalent electrical circuit) generated while applying an alternating current and a small voltage [52]. For biosensing, EIS is usually performed in the presence of a solution containing a redox probe such as potassium ferricyanide/ferrocyanide ([Fe(CN)6]^3−/4−^) to gather Faradaic current. When this is done, the electron transfer resistance (Ret) of the system is affected by the events occurring at the electrode’s surface (e.g., adsorption of biomolecules) [53]. Therefore, EIS has been used by researchers to investigate electrode modifications, such as antibody immobilization, as well as their recognition reactions with their antigens (or target analytes) y one hour, while the assay time of our EIS immunosensor is 40 min. Among the several EIS immunosensors described for CEA, only in the study [43,54,55]. One of the advantages of impedimetric biosensors over amperometric and voltametric biosensors is the use of a small amplitude voltage (˂10 mV) that makes them non-destructive devices. This means impedimetric biosensors can measure the analyte concentration without significantly disturbing the biomolecular probe layer done by Pan [56,57]. Recently, impedimetric biosensors have attracted much attention due to their various advantages, such as label-free detection, cost effectiveness, robustness, no need of expensive reagents, portability, and easiness of operation with no need of skilled users. However, they have not yet been fully exploited in commercial devices [55,58,59].

In this work, a novel EIS-based sensing platform has been developed for the early diagnosis of lung cancer via detecting and quantifying CEA and NSE as disease biomarkers. The sensing platform comprised magnetic manipulation, screen-printed gold electrodes (SPGE) and magnetic nanobeads (MB). MB were functionalized with antibodies (Ab) to bind the analyte in the sample. The MB–Ab–CEA and MB–Ab–NSE complexes were then moved over the sensing surface, where CEA and NSE capture antibodies were immobilized for affinity binding interactions. MB manipulation was used here as a microfluidic system to increase the chance of antigen–antibody complex formation, reducing diffusion time and allowing the separation of the biomarkers from the complex sample matrix prior to their detection and quantification.

## 2. Materials and Methods

An extended version of the materials and methods and results described in this manuscript can be found in the PhD thesis of the first author, accessible through the Cranfield University repository system (CERES). The link to CERES can be found in the “Data availability statement”.

### 2.1. Materials

The following analytical grade reagents were used for the experiments and were all purchased from Sigma-Aldrich (Dorset, UK): potassium hexacyanoferrate(II) trihydrate and potassium hexacyanoferrate(III), which were used as redox probe, phosphate buffer saline tablet (PBS) (0.01 M phosphate buffer, pH 7.4), sterile-filtered human serum, potassium hydroxide, potassium phosphate monobasic, sodium chloride, 1-3-dimethylaminopropyl-3-ethylcarbodiimide hydrochloride (EDC), N-hydroxysuccinide (NHS), cysteamine hydrochloride, ethanolamine, N,N-dimethyl formamide (DMF), 1,4-phenylene diisothiocyanate (PDITC), bovine serum albumin (BSA), pyridine, sulfuric acid, turboBeads™ carboxy (MB) and tween 20. Mouse monoclonal 12-140-01 and 12-140-10 anti-CEA antibodies, mouse monoclonal 10-7937-FIT and 10-7938-FIT anti-NSE antibodies, native human CEA protein, and purified native human NSE protein were from Fitzgerald Industries International (Acton, MA, USA). DropSens, DRP-220BT SPGE, were acquired from Metrohm (Cheshire, UK).

### 2.2. EIS Measurements

Electrochemical impedance spectroscopy measurements were performed using a PalmSens3 potentiostat (PalmSens BV, Houten, The Netherlands). The PalmSens was connected to a computer with the dedicated PStrace 4.8 software (PalmSens BV, Houten, The Netherlands), which was used to command the instrument and analyze the data. All EIS measurements were performed in a Faraday cage using a potential of 0.12 V and by applying a potential amplitude of 0.01 V, a current range between 10 µA and 10 mA, a frequency range between 50 kHz and 0.1 Hz, and a frequency number of 51. Measurements were performed by dispensing the redox probe (50 µL of 10 mM [K_3_Fe(CN_6_)]/[K_2_Fe(CN_6_)] prepared in 10 mM PBS pH 7.4).

EIS spectrum analyzer software PStrace 4.8 e was used to plot the Nyquist graphs. After fitting the graphs onto the Randles equivalent circuit, the software enabled the estimation of the resistance of the electron transfer (R_et_) for data analysis.

### 2.3. Functionalization of Magnetic Beads (MB) with Antibodies

For the functionalization of the magnetic beads (MB), 15 mg of MB were added to 400 µL of coupling buffer (potassium phosphate monobasic with sodium chloride) in a 2 mL centrifuge tube and were dispersed by ultra-sonication for 1 min using a SONIC 6MX ultrasonic bath (James Products Europe, Newton, UK). A washing step was then performed by separating the MB using a magnetic rack (Invitrogen, Waltham, MA, USA) and discarding the supernatant. The step was then repeated by the addition of the coupling buffer (400 µL) to the MB, their dispersion using the ultrasonic bath for 1 min, and separation by the magnetic rack. After the final washing step, the separated MB were resuspended in 400 µL of coupling buffer, and a solution of EDC/NHS (400 µL of EDC plus 400 µL NHS) was added. The magnetic beads were then left to agitate under a slow tilt rotation for 20 min. The MB were again separated using the magnetic rack, and the supernatant with unreacted reagents was discarded. Next, the coupling buffer (400 µL) containing 10 µL of 2.4 mg/mL of monoclonal antibodies (12-140-10 anti-CEA or 10-7938-FIT anti-NSE Ab) was added to the beads, and the mixture was incubated for 30 min while it was gently shaking on a slow tilt rotation. This was followed by three washes with a “standard” buffer consisting of PBS with 0.1% BSA and 0.05% Tween 20 to remove unbound antibodies. In the last step, the buffer was removed from the tube, and the MB functionalized with antibodies (MB–Ab) were resuspended in 400 µL of standard buffer, aliquoted, and stored in the fridge until use.

### 2.4. Biosensors Preparation

**Figure 1:**
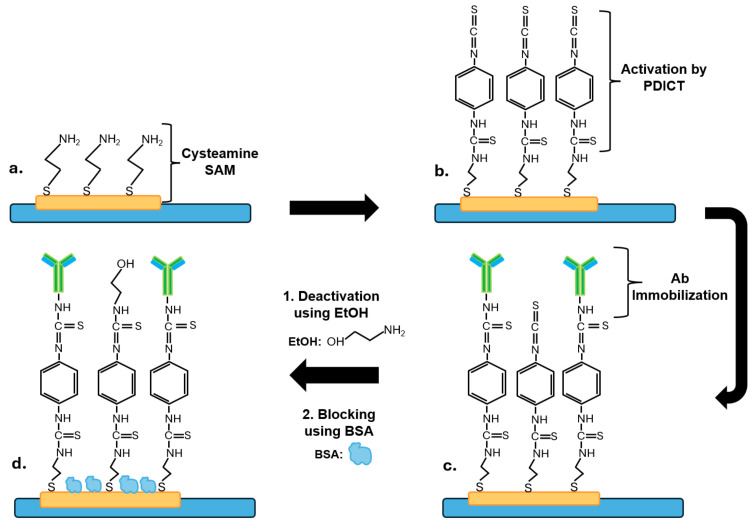
A scheme for the preparation of the biosensors (not at scale). (**a**) Formation of a cysteamine SAM on the gold working electrode; (**b**) activation of amino groups of the SAM using PDICT; (**c**) covalent immobilization of anti-CEA or anti-NSE monoclonal antibodies; (**d**) deactivation of unreacted thiocyanate functional groups using EtOH followed by the blocking of sensor’s surface by BSA.

An initial washing step was applied to all DRP-220BT SPGE before their use. The step consisted of dipping the electrodes in a solution of 50 mM potassium hydroxide with 25% hydrogen peroxide for 10 min, followed by rinsing with deionized water and drying with nitrogen gas. The biosensors were then prepared according to the steps described below and summarized in Figure 1.

The washed SPGE were placed in a petri dish on top of a wet tissue, and 10 mM cysteamine hydrochloride (10 µL) was dispensed on the working electrode (WE) and incubated at room temperature for 16 h to allow the formation of a self-assembled monolayer (SAM) on the sensors’ surface (Figure 1a). The SPGE were then washed three times with PBS and dried with nitrogen gas.

To activate the amino groups of the SAM, an activation solution (10 µL of PDITC in pyridine and DMF [*v*/*v* 1:9]) was dispensed on the WE and incubated at room temperature for 30 min (Figure 1b). The chemical PDITC, which is a homobifunctional crosslinking reagent, was selected as an activating agent as it is known for its stability and flexibility [56]. The activation step was followed by washing the electrodes three times with DMF and PBS before drying them with nitrogen gas.

Then, solutions (20 µL) of 10 µg/mL of anti-CEA 12-140-10 or anti-NSE 10-7937-FIT antibodies prepared in PBS were dispensed on the WE and incubated for two hours at room temperature (Figure 1c). After this, three washes with 50 µL of PBS to eliminate unbound antibodies from the surface were carried out. Next, a solution (20 µL) of 0.1 M ethanolamine (EtOH, pH 7.6) was then added on WE for 30 min (to deactivate unreacted thiocyanate terminal groups, Figure 1d), followed by washing the electrodes three times with 50 µL of PBS. Then, the sensors were incubated for 30 min in 1% BSA solution (100 µL) to block the surface and minimize non-specific binding (Figure 1d), and they were rinsed with PBS. The immobilization method of antibodies on a gold surface using the formation of a cysteamine SAM layer was adapted from Elshafey and colleagues [60].

### 2.5. CEA/NSE Immunosensors Development

**Figure 2:**
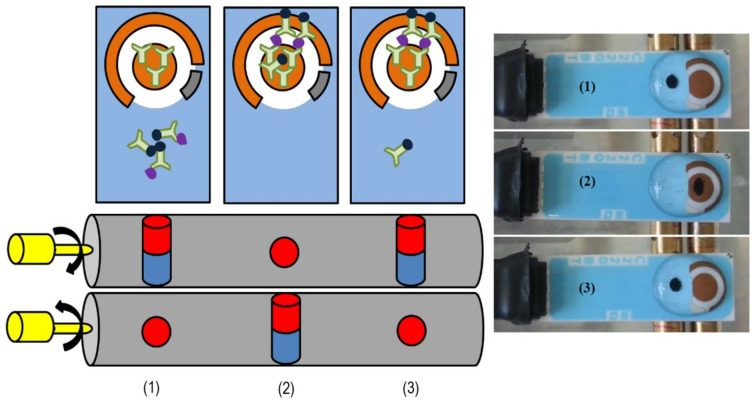
A scheme of the immunosensors platform is shown on the left. The photoshoots on the right show the movements of the MB; after capturing the analyte with functionalized MBs, these were added to the non-sensing area of SPGE, where the platform magnet was perpendicular to the electrode (1). The rotation of magnetic cylinders causes movement of MBs to the WE for analyte detection (2) and pull back the unbounded MB from the WE (3).

The sensing platform consisted of two magnets, which were enclosed in two rotatable cylinders. The rotation of the two cylinders allowed changing the polarity of the magnets, enabling the movement of the MB on the surface of SPGE, from the non-sensing area to the WE and vice-versa. Figure 2 is an illustration of the platform principle.

After preparing a range of concentrations of CEA or NSE, 45 µL of each concentration of analyte was mixed in a tube with 5 µL of MB–Ab, vortexed for a few seconds, and incubated for 20 min to allow the formation of the MB–Ab–analyte complex. The tube content was then washed three times with 50 µL of PBS using the magnetic rack for bead separation. The washed MB–Ab–analyte was redispersed in 50 µL of PBS and placed on the ceramic (non-sensing) part of SPGE, with the platform magnet pole facing up. The MB–Ab–analyte complex was then pulled to the sensing area (WE with Ab) by rotation of the magnetic bars, where it was incubated for 20 min. Next, the magnetic bars were rotated again to remove the unbounded MB–Ab from the WE surface. The SPGE was rinsed with PBS and dried with nitrogen gas before measuring the EIS signal, as explained in Section 2.2. The obtained Ret (Ω) values were calculated as a percentage of the blank signal (recorded in the absence of analyte) using the following equation: Ret_s_-Ret_0_/Ret_0_ × 100 (where Ret_s_ is the signal recorded for the sample and Ret_0_ is the signal obtained for the blank) and were expressed as %ΔR_et_, thus normalizing the sensor response, as reported previously in our research group [61].

## 3. Results

### 3.1. Immunosensor Optimization

Several optimizations of the immunosensors were conducted to obtain high sensitivity and specificity. For these experiments, the immunosensors were prepared and tested with various NSE or CEA concentrations by the formation of a sandwich sensor on top of the functionalized SPGE. The EIS signals were then recorded, the R_et_ values were evaluated using the PStrace software, and the %∆R_et_ for each concentration was calculated as explained in the Materials and Methods, in Section 2.3. As shown by the results, the attachment of the MB–Ab–analyte to the antibodies immobilized on the SPGE reduces the electron transfer from the redox probe to the electrode, thus increasing the R_et_ values proportionally to the concentrations of the analyte.

**Figure 3:**
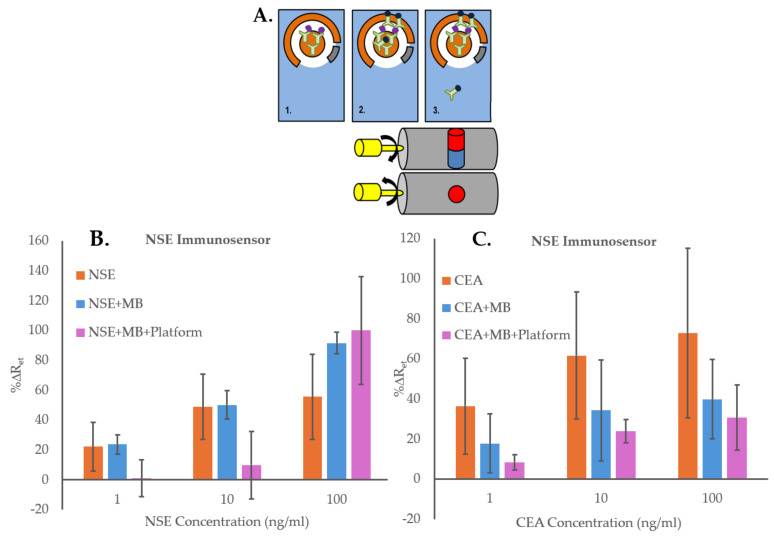
(**A**) Schematic illustration of the three assays formats used to demonstrate the advantage of using MB and the magnetic Platform: (**1**) Direct measurements of analyte concentrations, “NSE” (or “CEA”); (**2**) Measurements of analyte concentrations using MB–Ab (“NSE + MB” or “CEA + MB”); (**3**) Measurement of analyte concentrations using MB–Ab and the magnetic Platform (“NSE + MB + Platform” or “CEA + MB + Platform”). (**B**) NSE immunosensors responses to concentrations of specific analyte NSE (0–100 ng/mL) with experiments performed using the three different assay formats; (**C**) NSE immunosensors responses to concentrations of non-specific analyte CEA (0–100 ng/mL) with experiments performed using the three different assay formats. The error bars represent standard deviations of triplicates carried out with three immunosensors.

Before starting the optimization of the immunosensors, initial experiments were carried out to confirm the advantage of using the MB and the magnetic platform for the detection and quantification of biomarkers. These tests were performed using NSE as the target analyte, CEA as the interferent compound, and SPGE immunosensors for NSE, prepared as explained in Section 2.4. Three immunosensor formats (Figure 3A) were then explored: (1) solutions of NSE or CEA (0, 1, 10, 100 ng/mL) were incubated directly for 20 min on NSE immunosensors (“NSE”). The electrodes were then rinsed with PBS, dried, and their EIS signal recorded; (2) MB functionalized with anti-NSE Ab (MB–Ab) were first incubated for 20 min with solutions of NSE or CEA (0, 1, 10, 100 ng/mL) to obtain MB–Ab–analyte; then these were dispensed on the NSE immunosensors and after an incubation of 20 min, the electrodes were washed with PBS, dried and their EIS signal recorded (“NSE + MB”); (3) MB functionalized with anti-NSE Ab (MB–Ab) were first incubated for 20 min with solutions of NSE or CEA (0, 1, 10, 100 ng/mL) to obtain MB–Ab–analyte; then these were dispensed on the NSE immunosensors with the magnetic cylinders in a position concentrating the MB outside the sensing area. The magnetic cylinders were then rotated to move the MB–Ab–analyte (and free MB–Ab) to the NSE immunosensor area. After an incubation of 20 min, the magnetic cylinders were then rotated again to move the unbounded MB–Ab back to the SPGE non-sensing area. The electrodes were then washed with PBS, dried, and their EIS signals recorded (“NSE + MB + Platform”).

**Figure 4:**
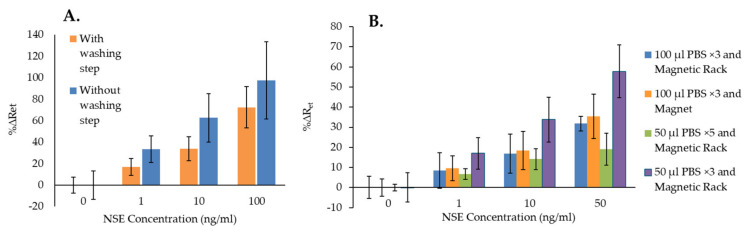
(**A**) Comparison of NSE immunosensor responses with and without applying a washing step after mixing MB–Ab and the analyte before incubation on SPGE–Ab surface; (**B**) the comparison of NSE immunosensor responses with various washing methods before incubation on SPGE–Ab surface. The error bars represent the standard deviation of triplicates.

The results of the experiments using NSE as the specific analyte, presented in Figure 3B, show that the NSE immunosensor could detect the biomarker directly (“NSE”), as the sensors’ signals increased proportionally to the concentration of the analyte. However, when using the MB–Ab, “NSE + MB”, the immunosensor responses were higher, probably thanks to the formation of sandwich assays on the SPGE. Nevertheless, the highest changes in %ΔRet values were obtained with the use of MB and the platform, “NSE + MB + Platform”, most likely because the magnetic field enabled bringing the MB–Ab–NSE closer to the sensor surface, increasing the chance of the anti-NSE antibodies on the SPGE to capture the specific analyte. The EIS spectra used for Figure 3B are shown in Figures S1–S3. To investigate the effect of using the MB–Ab and the platform on the biosensor’s non-specific binding, the experiments were repeated by testing the non-specific analyte, CEA, with the NSE immunosensors and using the three sensing formats. The results reported in Figure 3C show that the highest non-specific responses were obtained by adding CEA samples directly to the sensing area, “CEA”, demonstrating that with this sensing format, the blocking of the SAM layer on the sensor surface was insufficient. The sensor responses to various concentrations of CEA decreased when MB–Ab were used for the testing (“CEA + MB”), demonstrating that the sandwich assay format helped in reducing the non-specific interactions. Nevertheless, the lowest EIS responses were obtained using both MB–Ab and the proposed platform, “CEA + MB + Platform”. This might be due to the ability of the magnetic field to remove, in the last step, any free MB–Ab, even those with loosely attached CEA, which would then be unable to adsorb non-specifically on the sensor’s surface. The EIS spectra used for Figure 3C are shown in Figures S4–S6. The direct comparison between the specific and non-specific responses of the NSE immunosensors obtained when using NSE + MB + Platform, reported in Figure S7, shows that the immunosensors’ signals increased while increasing the concentration of both NSE and CEA. However, significantly higher %ΔR_et_ values were obtained for the specific analyte, demonstrating the advantage of using the MB and the platform. Nonetheless, the increase in signal seen for the non-specific analyte combined with the large standard deviations obtained when testing NSE demonstrated that the immunosensors required a full optimization for improved performance. Therefore, the first important optimization, performed using NSE as analyte, was the inclusion of a washing step after formation of the MB–Ab–analyte complex. For this, the complex was first precipitated using a magnetic rack and was washed three times. It was then redispersed in buffer and added to the functionalized SPGE on the magnetic platform. The EIS results obtained with and without performing the washing step prior to incubation of the MB–Ab–analyte on the SPGE surface are presented in Figure 4A. The figure shows that the application of the washing step, although it reduces the overall immunosensor signal, it also decreases the standard deviation of the measurements, hence increasing the accuracy of the sensors’ responses. Figure 4B shows the optimization of the washing volume (50 or 100 µL of PBS buffer), the number of repeats, and the optimization of the type of magnet used to precipitate the MB–Ab–analyte among repeated washings.

Figure 4B shows that washing the MB–Ab–analyte complex with 50 µL of PBS for 5 times (green bar) resulted in the lowest sensor signals with the smallest error bars in comparison with the other types of washing methods. Applying a washing step 5 times is likely to start removing the analyte bound to the MB–Ab, decreasing the overall immunosensor’s response. This is also confirmed by the fact that the highest signals were obtained when washing the MB–Ab–analyte with the smallest volume, 50 µL of PBS, for only 3 times. Similarly, when washings were performed with 100 µL of PBS, lower %∆R_et_ values were obtained in comparison with 50 µL of PBS, confirming that the higher volume of washing solution starts removing the analyte from the MB–Ab. Figure 4B also shows that similar error bars were obtained for all types of washings except when using 50 µL of PBS for 5 times, although this generated the lowest sensor signal. However, the lowest coefficients of variation (%CV) were obtained when washing was performed with 50 µL of PBS for 3 times. This also produced the highest sensor response, especially when using a magnetic rack rather than a single magnet for the isolation of the MB. Therefore, such washing protocol was selected as the best and applied to all subsequent experiments for both NSE and CEA immunosensors.

**Figure 5:**
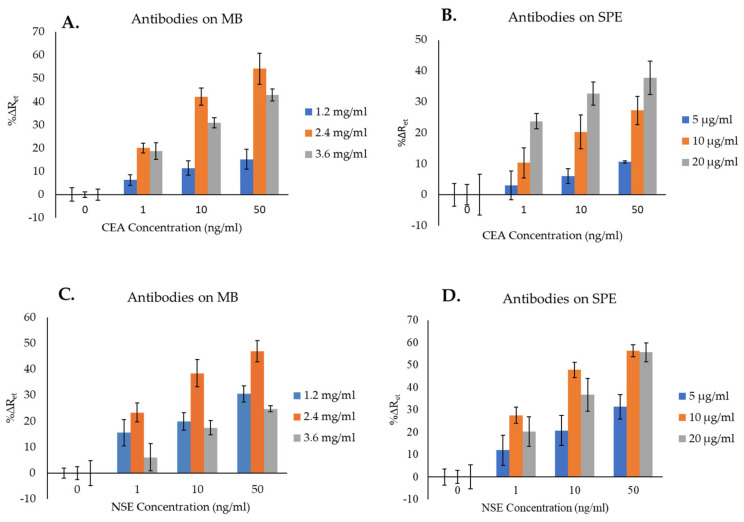
Optimization of the concentration of antibodies to attach to both MB and SPGE surface for the detection of CEA (**A**,**B**) and NSE (**C**,**D**). The error bars represent the standard deviations of triplicates.

Other optimizations included optimizing the antibody concentration to attach to the MB (Figure 5A,C) for both CEA and NSE and the selection of the best concentration of Ab for the immobilization on the surface of SPGE for both analytes (Figure 5B,D).

Figure 5A shows the changes in impedance signals (%∆R_et_) obtained by increasing the CEA antibody attached to the MB. The lowest sensors’ signals were obtained when MB were functionalized with 1.2 mg/mL of antibody. As the antibody’s concentration increased from 1.2 to 2.4 mg/mL, higher %∆R_et_ values were achieved. The reason for this can be attributed to a higher chance for the analyte to be captured by the MB–Ab. Further increase in the Ab concentration on the MB from 2.4 to 3.6 mg/mL resulted in a reduction in %∆R_et_, which can be attributed to steric hindrance, as the presence of too many Ab molecules on the surface of MB can hinder the attachment of the analyte. Therefore, from the tested concentrations (1.2, 2.4, and 3.6 mg/mL), 2.4 mg/mL was chosen as the optimum and used for further experiments.

Figure 5B, depicting the optimization of anti-CEA Ab to immobilize on the SPGE surface, shows that the lowest signals were obtained by immobilizing 5 µg/mL of Ab. As the concentration of Ab immobilized on the immunosensor’s surface increased (i.e., 10 and 20 µg/mL), higher %∆R_et_ signals were obtained, with the highest values recorded using 20 µg/mL of Ab, suggesting this was the optimum concentration among those tested. Although an even higher Ab’s concentration might have resulted in higher sensors’ signals, this was not tested as it would make the immunosensors too expensive. Therefore, 20 µg/mL was chosen as the optimum concentration of anti-CEA Ab to immobilize on the sensor surface and was used for further experiments.

Regarding the optimization of the NSE immunosensors, Figure 5C shows that, similarly to CEA, increasing the concentration of anti-NSE antibody on the surface of MB from 1.2 mg/mL to 2.4 mg/mL resulted in an increment of the %∆R_et_ signals, whereas the highest Ab concentration tested (3.6 mg/mL) caused a reduction in the immunosensor signals due to steric hindrance. The highest %∆R_et_ signals were therefore obtained using MB functionalized with 2.4 mg/mL of Ab, and this was regarded as optimum and selected for further experiments.

Figure 5D, presenting the optimization of the anti-NSE Ab concentration for its immobilization on the SPGE surface, shows that the lowest signals were obtained by preparing immunosensors with 5 µg/mL of antibody. As the concentration of Ab increased to 10 and 20 µg/mL, higher %∆R_et_ were obtained. The NSE immunosensors prepared by immobilizing 10 µg/mL of anti-NSE Ab achieved the highest %∆R_et_ signals for the detection of NSE in comparison with the other two Ab concentrations. Thereby, 10 µg/mL was chosen as the optimum concentration of anti-NSE Ab to immobilize on the SPGE and was used for further experiments.

Once the immunoassays were fully optimized, the resulting immunosensors were used for the quantification of CEA and NSE, both in buffer and human serum, and to detect NSE or CEA as interfering analytes to evaluate the immunosensors cross-reactivity.

### 3.2. CEA and NSE Quantification in Buffer 

**Figure 6:**
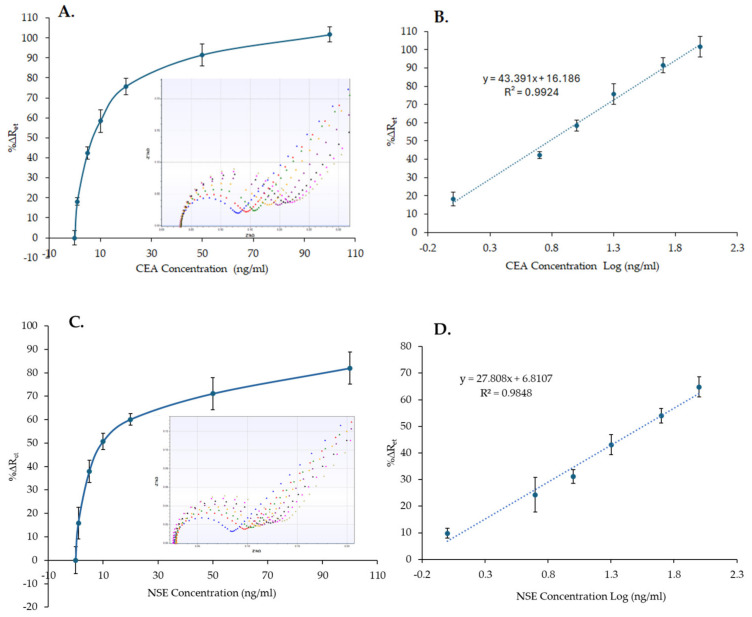
Nonlinear calibration plot (**A**,**C**) and linear range (**B**,**D**) of the CEA and NSE immunosensors, respectively, versus CEA and NSE concentrations (1–100 ng/mL) in PBS. The insets in (**A**,**C**) show examples of the EIS spectra obtained for the calibration curves (functionalized SPGE (blue), 0 ng/mL (red), 1 ng/mL (green), 5 ng/mL (yellow), 10 ng/mL (purple), 20 ng/mL (black), 50 ng/mL (pink), 100 ng/mL (khaki). The error bars represent the standard deviations of measurements performed with three sensors.

The CEA and NSE immunosensors were tested by measuring increasing concentrations of protein samples in PBS to generate a standard curve. After mixing CEA or NSE samples with MB–Ab and after performing the optimized washing step to remove unbound reagents, the MB–Ab–analyte complexes were measured by dispensing them onto the CEA and NSE immunosensor and recording the signals by EIS. The standard curves (Figure 6A,C) were created by plotting the averaged %∆R_et_ values, recorded using three sensors, versus the concentration of CEA or NSE. As Figure 6A,C shows, increasing the concentration of CEA and NSE results in an increased %∆R_et_ signal, proportional to the quantity of analytes present in the sample. Whereas the insets in Figure 6A,C shows the EIS spectra recorded for one sensor, all the spectra obtained and used for the calibration curves are reported in Figures S8 and S9 for CEA and NSE, respectively.

The linear range of the CEA and NSE immunosensors in PBS was obtained by plotting the sensor responses (%∆R_et_) versus the concentration of CEA or NSE in a logarithmic scale, Figure 6B,D. The limit of detection (LoD) was then determined as the concentrations equal to 3 times the standard deviation of the blank response (R_et_ in the absence of the analyte) divided by the slope of the linear curve. LoD values of 0.26 ng/mL and 0.18 ng/mL with correlation coefficients of 0.9924 and 0.9848 were obtained for CEA and NSE, respectively.

### 3.3. CEA and NSE Quantification in Serum

**Figure 7:**
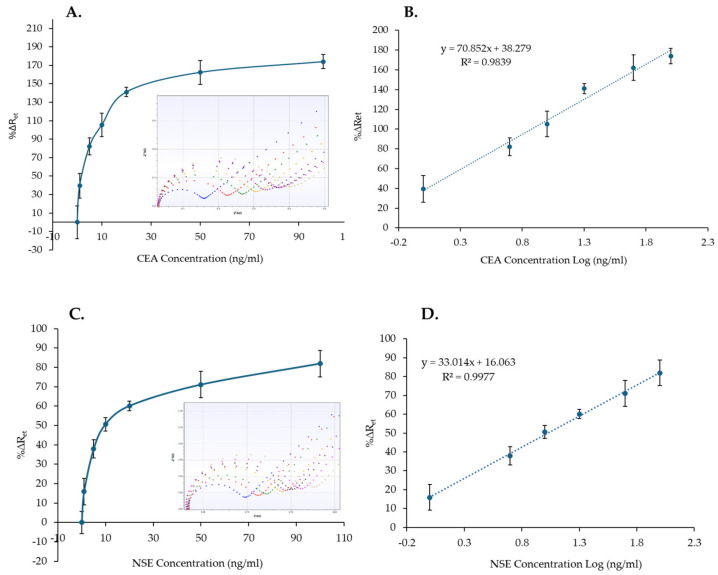
Nonlinear calibration plot (**A**,**C**) and the linear responses (**B**,**D**) of the CEA and NSE immunosensors versus CEA and NSE concentrations (1–100 ng/mL) in serum. The insets in (**A**,**C**) show a examples of the EIS spectra obtained for the calibration curves (functionalized SPGE (blue), 0 ng/mL (red), 1 ng/mL (green), 5 ng/mL (yellow), 10 ng/mL (purple), 20 ng/mL (black), 50 ng/mL (pink), 100 ng/mL (khaki). The error bars represent standard deviations of measurements performed with three sensors.

The response of CEA and NSE immunosensors to a range of protein concentrations (0–100 µg/mL) spiked in 100% human serum was tested. The MB–Ab were used to capture the analyte from the serum sample and to move it via the magnetic platform onto the functionalized SPGE surface to complete the sandwich immunosensors. The standard curves (Figure 7A,C) were then created, using three sensors, based on the calculated %∆R_et_ against the concentration of CEA or NSE. Similarly to the immunosensor responses in PBS, the %∆R_et_ signal increased by increasing the concentration of the analytes in serum (Figure 7A,C). Whereas the insets in Figure 7A,C show the EIS spectra recorded for one immunosensor, all the spectra obtained and used for the calibration curves are reported in Figures S10 and S11 for CEA and NSE, respectively.

The %∆R_et_ and the CEA or NSE concentrations in logarithmic scale were used to plot the linear range of CEA and NSE immunosensors (Figure 7B,D). Linear curves with correlation coefficients of 0.9839 and 0.9977 and LoD values of 0.76 ng/mL and 0.52 ng/mL were obtained for CEA and NSE, respectively. Although the achieved LoDs are higher than the LoDs for both analytes in PBS, which were 0.26 ng/mL and 0.18 ng/mL, the values are lower than the clinical cut-off values of 5 ng/mL and 12 ng/mL for CEA and NSE, respectively, and are, therefore, clinically relevant.

### 3.4. Cross-Reactivity Evaluation

**Figure 8:**
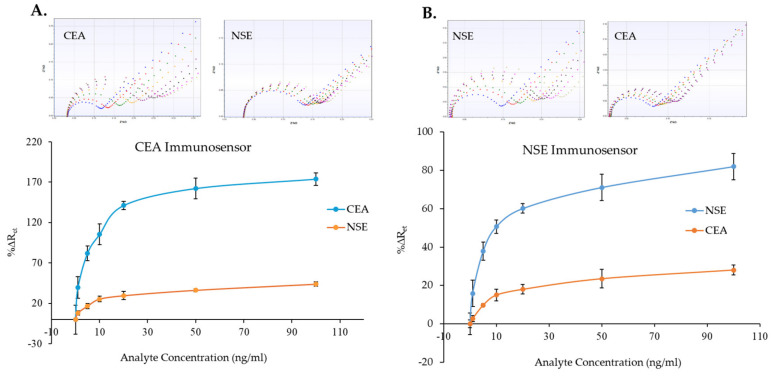
Cross-reactivity study: CEA (**A**) and NSE (**B**) immunosensors responses to various concentration of specific (CEA and NSE) and non-specific (NSE and CEA) analytes. Examples of EIS spectra for specific and non-specific analyte obtained with both immunosensors are shown above the calibration curves (functionalized SPE (blue), 0 ng/mL (red), 1 ng/mL (green), 5 ng/mL (yellow), 10 ng/mL (purple), 20 ng/mL (black), 50 ng/mL (pink), 100 ng/mL (grey). The error bars represent the standard deviations of measurements performed with three sensors.

Since the immunosensors were able to detect CEA and NSE in both PBS and serum samples, cross-reactivity tests were performed to check whether the sensor signal generation was only due to binding of the molecule of interest. These experiments were carried out by measuring several CEA and NSE concentrations (0–100 ng/mL) spiked in 100% human serum with both the CEA and the NSE immunosensors. Figure 8A,B presents the results of the cross-reactivity tests. As shown by the comparison graphs, the CEA and NSE immunosensor responses did increase minimally with increments of the concentration of the non-specific analyte. Nevertheless, although the sensors responded to the non-specific analyte, the signals recorded were significantly lower than the specific sensor responses. For instance, the highest %∆R_et_ for NSE detection by the CEA immunosensor was 48.3% (Figure 8A), which was obtained when testing 100 ng/mL of NSE, while a %∆R_et_ value of 82.1% was achieved for 5 ng/mL of CEA. Therefore, from the sensor responses, it was possible to distinguish between 100 ng/mL of NSE and 5 ng/mL of CSA. Similarly, the highest %∆R_et_ for CEA detection using the NSE immunosensor was 28.0% (Figure 8B), which was achieved with 100 ng/mL of CEA, while a %∆R_et_ value of 37.9% was obtained when testing 5 ng/mL of NSE, enabling again to distinguish between 100 ng/mL of NSE and 5 ng/mL of CEA. Whereas examples of EIS measurements recorded for the two immunosensors for the specific and non-specific analytes are shown on top of Figure 8A,B, the full set of EIS spectra obtained during the cross-reactivity evaluation and used to plot the curves are shown in Figures S12 and S13 for the CEA and NSE, respectively.

According to the results obtained in this study, the developed NSE and CEA immunosensors with the use of MB and the proposed platform were successful in detecting specific analytes in both PBS and serum in clinically relevant ranges. Future work will further test and optimize the sensing platform and the magnetic manipulation concept with serum samples taken from patients and validated in clinical settings.

## 4. Discussion

Among the several lung cancer serum biomarkers that have been proposed in the literature, CEA and NSE are among the most promising. CEA is known as a reliable, sensitive, and specific biomarker of lung cancer with cut-off values of around 5 ng/mL. Similarly, NSE is known to be a reliable, sensitive, and specific biomarker for SCLC. Thus, it is an important biomarker for the detection of lung cancer and especially to distinguish between SCLC and NSCLC. The cut-off value for NSE in serum has been reported as 12 ng/mL.

The immunosensors developed here were prepared in a similar manner for both biomarkers, with the only difference being the monoclonal antibodies used to functionalize the MB to capture the analytes from the samples and the monoclonal antibodies immobilized on the SPGE to complete the sandwich immunosensors. By exploiting the novel combination of the MB and the magnetic platform and after performing a series of important assays’ optimizations, we have demonstrated quantification within 40 min of CEA and NSE proteins in pure serum well below the clinical cut-off values (LoD of 0.76 ng/mL and 0.52 ng/mL for CEA and NSE, respectively), with linearity in clinically relevant concentration ranges (1–100 ng/mL) for both immunosensors. In addition, the results obtained from the cross-reactivity experiments, performed in 100% serum, have shown minimal immunosensor responses for non-specific proteins, enabling discrimination between 5 ng/mL of the specific protein and 100 ng/mL of the non-specific ones. We envisage that the two biosensors could be used in the future for POCT in clinical settings (e.g., medical centers and hospitals) where a small amount of patients’ blood (<1 mL) could be withdrawn, filtered quickly to obtain serum [62] and dispensed on the immunosensors for biomarker’s quantification.

Examples of biosensors for CEA and NSE have already been reported in the literature. The biosensors for CEA and their performances are listed in Table 1, where our immunosensors is also reported for comparison. Similarly, the biosensors for NSE are listed and compared in Table 2.

Although the LoD values of the biosensors reported in Table 1 and Table 2 are in general lower than our biosensors, the vast majority were evaluated in buffer and not in undiluted serum. In most examples, testing in spiked serum (or diluted serum) samples was demonstrated with decent recoveries, but without discussing openly the effect of the complex matrix either on the LoD or on the linearity range of the biosensors. Another important advantage of our biosensors over others reported in Table 1 and Table 2 is the assay time. For instance, in the case of EIS biosensors for CEA, Hou and coworkers [54] reported an assay time of more than one hour, Zhou and colleagues [63] of exactly one hour, while the assay time of our EIS immunosensor is 40 min. Among the several EIS immunosensors described for CEA, only in the study done by Pan and Yang in 2007 [64] was CEA measured using functionalized MB. They were able to detect CEA in PBS with an incubation time of 30 min, achieving a LoD of 0.5 ng/mL, which is higher than the one obtained in this work (0.26 ng/mL). In addition, their sensing platform required higher amounts of reagents (e.g., PBS buffer) in comparison with our immunosensors. Among the other biosensor examples listed in Table 1, only Jin et al. (2014) [65] have reported detection of CEA with MB, even though they used cyclic voltammetry rather than EIS. Their measurements were performed in buffer, where they obtained a LoD of 5 ng/mL, which is higher than our immunosensor (0.26 ng/mL).

**Table 1:** Biosensors developed for CEA detection.

Type of Sensor	Linear Range	Limit of Detection (LoD)	Reference
Differential pulse voltammetry	0.001–100 pg/mL	0.001 pg/mL	[66]
	0.1–750 ng/mL	~90 pg/mL	[67]
	0.01–100 ng/mL	0.003 pg/mL	[68]
Amperometric	0.001–50 ng/mL	0.3 pg/mL	[69]
	0.1–2 ng/mL	60 pg/mL	[70]
Anodic stripping voltammetry	0.05–1000 pg/mL	0.024 pg/mL	[71]
Cyclic voltammetry	5–60 ng/mL	5 ng/mL	[65]
Electronic	0.25 pg/mL–800 µg/mL	0.25 pg/mL	[23]
Surface plasmon resonance	0.4–25 ng/mL	100 pg/mL	[24]
Electrochemiluminescence	5–300 ng/mL	2.51 ng/mL	[26]
Impedimetric	0.001–80 ng/mL	0.64 pg/mL	[54]
	0.05 pg/mL–20 ng/mL	0.023 pg/mL	[72]
	1.5–60 ng/mL	500 pg/mL	[64]
	0.5–20 ng/mL	100 pg/mL	[73]
	1–500 pg/mL 1–40 ng/ml	0.03 pg/mL	[74]
	0.001–100 ng/mL	0.1 pg/mL	[63]
	0.1–1000 ng/mL	60 pg/mL	[75]
**This work**	**1–100 ng/mL (serum)**	**0.76 ng/mL (serum)**	**-**

**Table 2:** Biosensors developed for NSE detection.

Type of Sensor	Linear Range	Limit of Detection (LoD)	Reference
Voltametric (differential pulse voltammetry, square wave voltammetry)	0–25 ng/mL	4.6 ng/mL	[46]
	1–150 ng/mL	0.9 ng/mL	[76]
	0.001–200 ng/mL	0.26 pg/mL	[77]
	0.001–100 ng/mL	0.0003 ng/mL	[48]
	0.01–100 ng/mL	0.003 ng/mL	[47]
Quantum dots based immunosensors	0.5–50 ng/mL	0.2 ng/mL	[44]
	0.001–100 ng/mL	0.02 pg/mL	[41]
	0.1 pg/mL–1000 ng/mL	0.09 pg/mL	[45]
Amperometric	0.01–100 ng/mL	0.0078 ng/mL	[38]
Field effect transistor	1–1000 ng/mL	100 ng/mL	[78]
Electrochemiluminescence	0.01 pg/mL–10 ng/mL	0.01 pg/mL	[79]
Plasmonic	0.17–1.7 µg/mL	21 ng/mL	[51]
Optical	5–125 ng/mL	12 ng/mL	[80]
	1–1000 ng/mL	N/A	[49]
	1–1000 ng/mL	0.05 ng/mL	[50]
Impedimetric	1–50 pg/mL	0.5 pg/mL	[42]
**This work**	**1–100 ng/mL (serum)**	**0.52 ng/mL (serum)**	-

As it can be observed in Table 2, whereas there are several studies reported in the literature that describe biosensors for the detection of NSE, the examples exploiting EIS are limited. Among all the studies reported, several have shown LoDs lower than the one obtained in our work, but as mentioned previously, most LoDs were assessed in buffer and not in serum. Among the examples reported in Table 2, only Barton and colleagues [42] have used EIS to measure NSE concentration. They have reported a linear detection range of 1–50 pg/mL in buffer and have achieved a LoD of 0.5 pg/mL. Nevertheless, it is not possible to fully compare their biosensor with ours, as they do not mention either the assay time or the amounts of reagents used for analyte detection.

## 5. Conclusions

Low-cost, disposable, and simple devices enabling the facile point-of-care quantification of serum cancer biomarkers would enable early detection of lung cancer as well as monitoring its treatment.

Therefore, in this work, CEA and NSE, which are known to be reliable, sensitive, and specific serum biomarkers for lung cancer, were selected as target analytes for the development of two biosensors. The resulting CEA and NSE immunosensors, described in this work, exploit magnetic beads functionalized with Ab to capture the biomarkers from serum samples and a magnetic platform to move the MB–Ab–biomarker complex on SPGE functionalized with a second Ab to complete the sandwich immunosensor, enabling electrochemical quantification by EIS. The detection of CEA and NSE with the use of MB and the proposed platform is novel and has not been reported elsewhere.

The results obtained in this study have demonstrated that the developed CEA and NSE electrochemical immunosensors were successful in quantifying within 40 min the two biomarkers in serum with LoDs of 0.76 ng/mL and 0.52 ng/mL, which are below the cut-off values of 5 ng/mL and 12 ng/mL for CEA and NSE, respectively. The immunosensors also showed linear responses in clinically relevant concentration ranges (0–100 ng/mL), demonstrating their potential to become powerful point-of-care screening devices leading to early diagnosis of lung cancer and enabling prompt therapeutic interventions with improved clinical outcomes.

## Data Availability

Data supporting this study are openly available from Cranfield University repository, CERES, at (https://dspace.lib.cranfield.ac.uk/handle/1826/19632, accessed on 16 November 2024).

## Supplementary Materials

The following supportive information can be downloaded at: https://www.mdpi.com/article/10.3390/bios14120624/s1, Figure S1: EIS spectra of ‘NSE’ immunosensors; Figure S2: EIS spectra of ‘NSE + MB’ immunosensors; Figure S3: EIS spectra of ‘NSE + MB + Platform’ immunosensors; Figure S4: EIS spectra for NSE immunosensors when testing CEA; Figure S5: EIS spectra of NSE immunosensor with MB when testing CEA; Figure S6: EIS spectra of NSE immunosensor, with MB and the magnetic platform when testing CEA; Figure S7: Comparison of NSE + MB + Platform immunosensors when testing NSE and CEA; Figure S8: EIS spectra of CEA immunosensors when testing increasing concentrations of CEA in buffer; Figure S9: EIS spectra of NSE immunosensors when testing increasing concentrations of NSE in buffer; Figure S10: EIS spectra of CEA immunosensors when testing increasing concentrations of CEA in serum; Figure S11: EIS spectra of NSE immunosensors when testing increasing concentrations of NSE in serum; Figure S12: EIS spectra of cross-reactivity study of CEA immunosensors; Figure S13: EIS spectra of cross-reactivity study of NSE immunosensor.
