# Supplementary material for: Impedimetric Biosensors for the Quantification of Serum Biomarkers for Early Detection of Lung Cancer
Source: Biosensors (Basel). 2024 Dec 18;14(12):624. doi: 10.3390/bios14120624 (PMC11674673; doi:10.3390/bios14120624)
Supplement: Supplementary file 1 [file biosensors-14-00624-s001.zip › biosensors-3316034-supplementary.pdf]

Supplementary Materials

Impedimetric Biosensors for the Quantification of Serum Biomarkers for Early Detection of Lung Cancer

Mahdi Arabnejad [1,2], Ibtisam E. Tothill [1,*] and Iva Chianella [1,*]

1 Faculty of Engineering and Applied Sciences, Cranfield University, Cranfield, Bedfordshire MK43 0AL, UK; mahdia@silveray.co.uk
2 Silveray, Stockport Road West, Stockport SK6 2BP, UK
* Correspondence: i.tothill@cranfield.ac.uk (I.E.T.); i.chianella.1998@cranfield.ac.uk (I.C.); Tel.: +44-(0)-1234-758322 (I.C.)

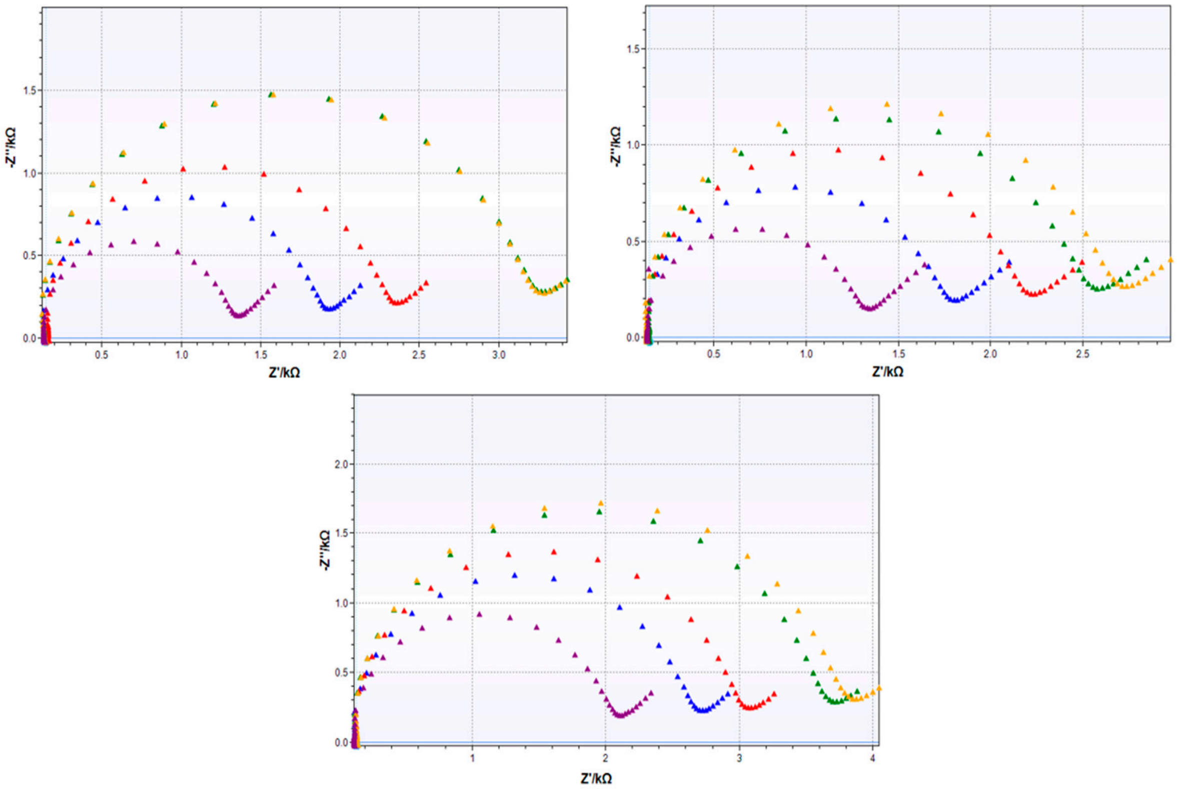

Figure S1. EIS spectra (Nyquist plots) of the three NSE immunosensors tested by direct incubation of increasing concentrations of the specific analyte, NSE, 'NSE'. Legend of EIS spectra: functionalized SPE (Purple), 0 ng/ml NSE (Blue), 1 ng/ml NSE (Red), 10 ng/ml NSE (Green), 100 ng/ml NSE (Yellow).

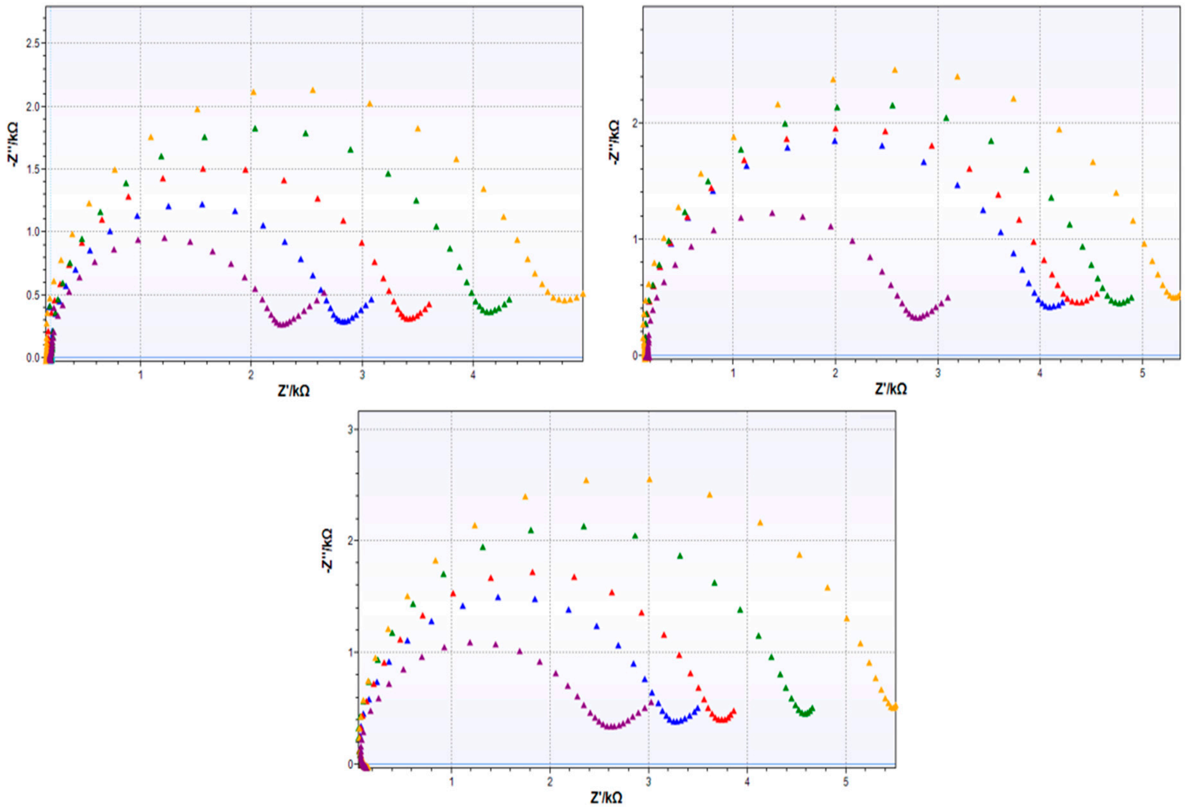

Figure S2. EIS spectra (Nyquist plots) of three NSE immunosensors tested by incubating MB-Ab with increasing concentrations of the specific analyte (NSE) followed by dispensing and incubating the resulting complex on the SPGE sensors ('NSE+MB'). Legend of EIS spectra: functionalized SPE (Purple), 0 ng/ml NSE (Blue), 1 ng/ml NSE (Red), 10 ng/ml NSE (Green), 100 ng/ml NSE (Yellow).

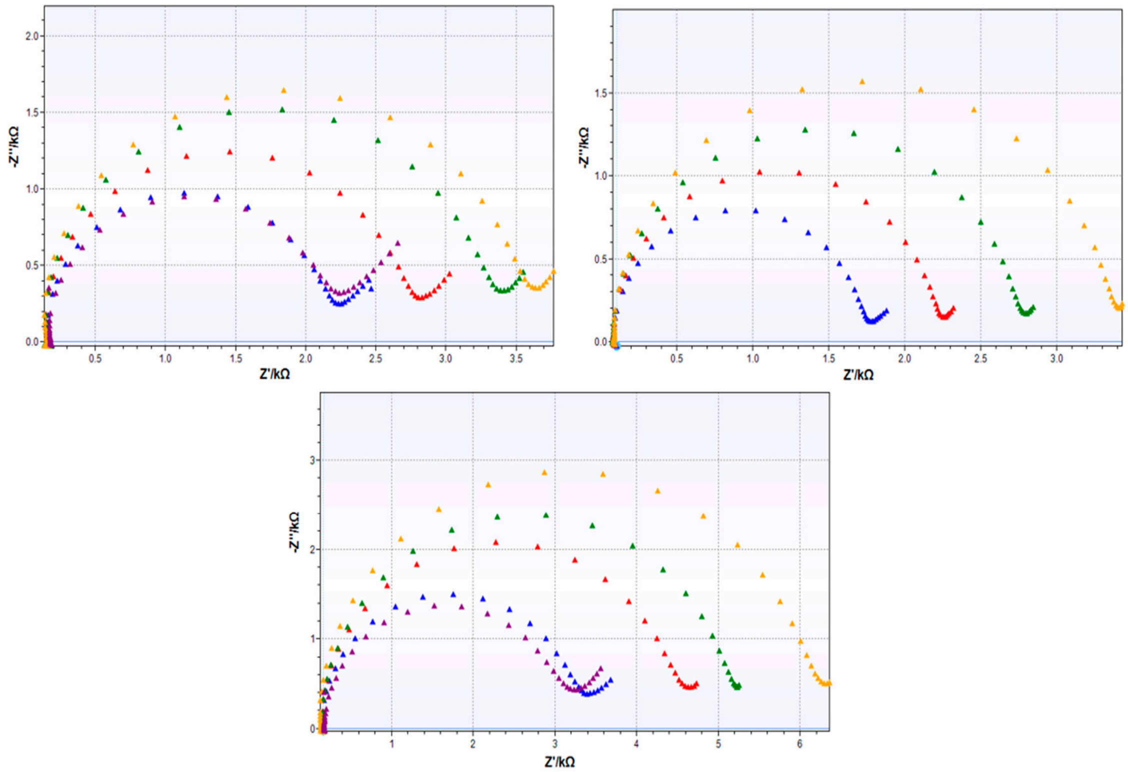

Figure S3. EIS spectra (Nyquist plots) of three NSE immunosensors tested incubating MB-Ab with increasing concentrations of the specific analyte (NSE) followed by dispensing and incubating the resulting complex on the SPGE with the magnetic platform, (enabling to move samples in and out the immunosensing area, 'NSE+MB+Platform'). Legend of EIS spectra: functionalized SPE (Purple), 0 ng/ml NSE (Blue), 1 ng/ml NSE (Red), 10 ng/ml NSE (Green), 100 ng/ml NSE (Yellow).

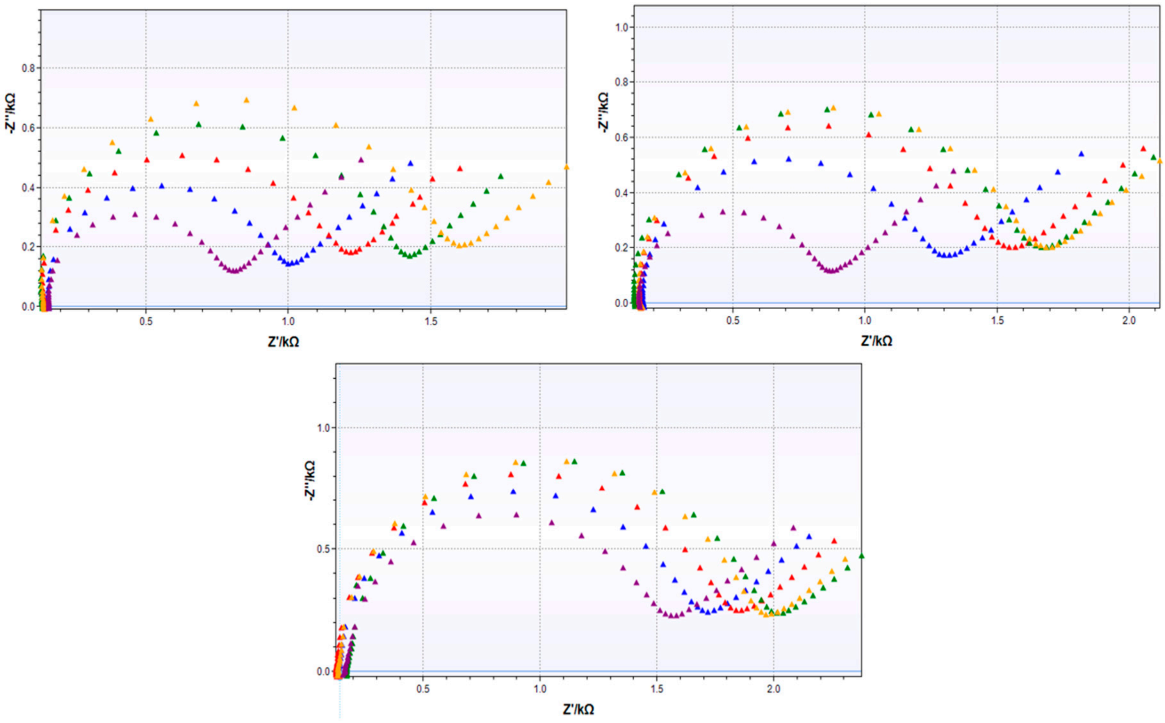

Figure S4. EIS spectra (Nyquist plots) of the three NSE immunosensors tested by direct incubation of increasing concentrations of the non-specific analyte, CEA ('CEA'). Legend of EIS spectra: functionalized SPE (Purple), 0 ng/ml NSE (Blue), 1 ng/ml NSE (Red), 10 ng/ml NSE (Green), 100 ng/ml NSE (Yellow).

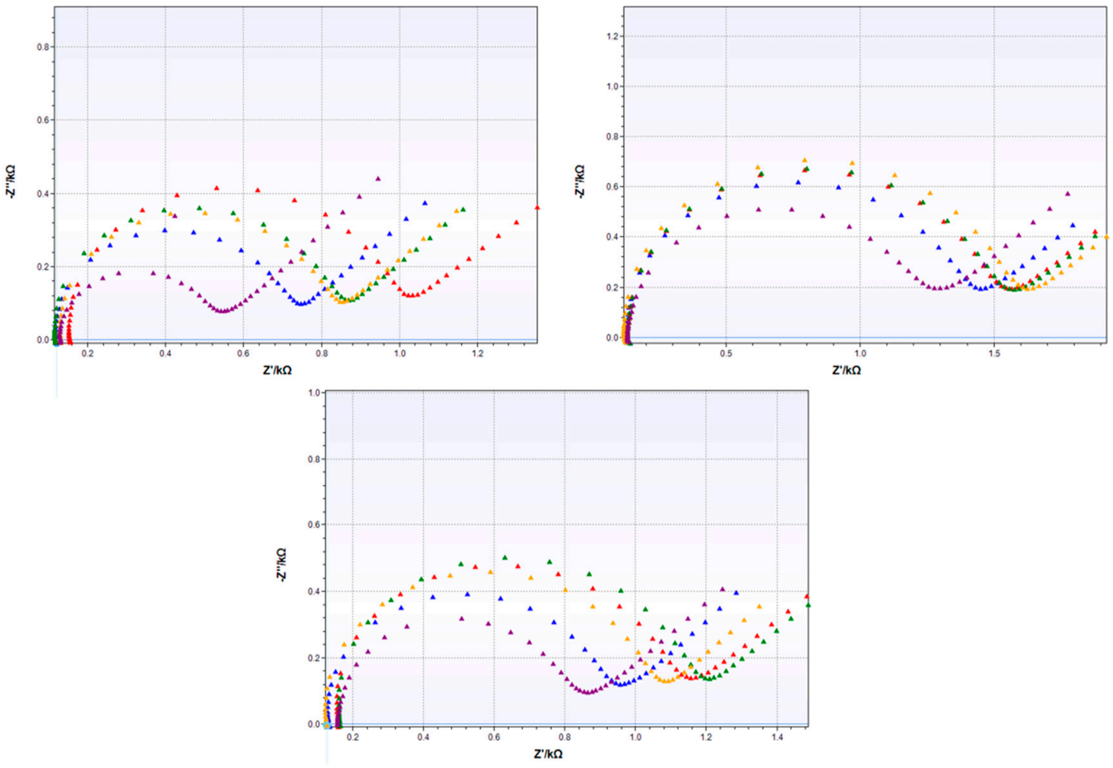

Figure S5. EIS spectra (Nyquist plots) of three NSE immunosensors tested by incubating MB-Ab with increasing concentrations of the non-specific analyte (CEA) followed by dispensing and incubating the resulting complex on the SPGE sensors ('CEA+MB'). Legend of EIS spectra: functionalized SPE (Purple), 0 ng/ml NSE (Blue), 1 ng/ml NSE (Red), 10 ng/ml NSE (Green), 100 ng/ml NSE (Yellow).

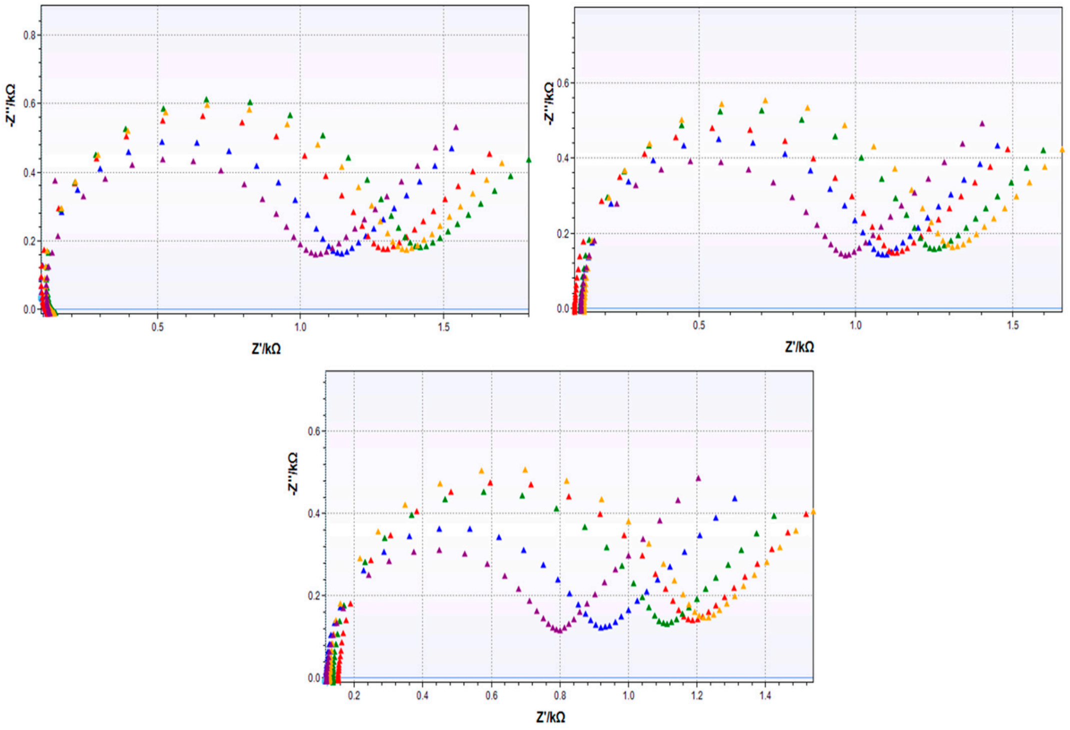

Figure S6. EIS spectra (Nyquist plots) of three NSE immunosensors tested incubating MB-Ab with increasing concentrations of the non-specific analyte (CEA) followed by dispensing and incubating the resulting complex on the SPGE with the magnetic platform, (enabling to move samples in and out the immunosensing area, 'CEA+MB+Platform'). Legend of EIS spectra: functionalized SPE (Purple), 0 ng/ml NSE (Blue), 1 ng/ml NSE (Red), 10 ng/ml NSE (Green), 100 ng/ml NSE (Yellow).

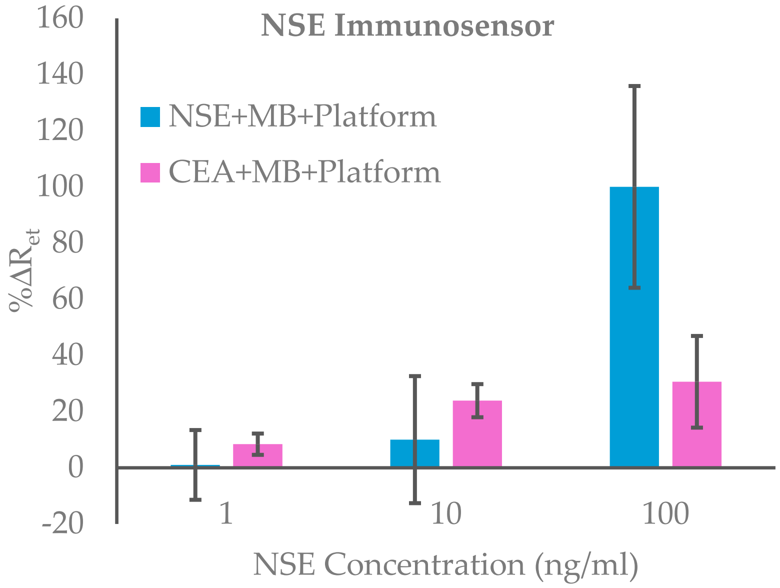

Figure S7. Comparison of specific (blue) and non-specific (pink) responses of NSE immunosensors when using the MB-Ab and the magnetic platform. The error bars represent the standard deviations of triplicates.

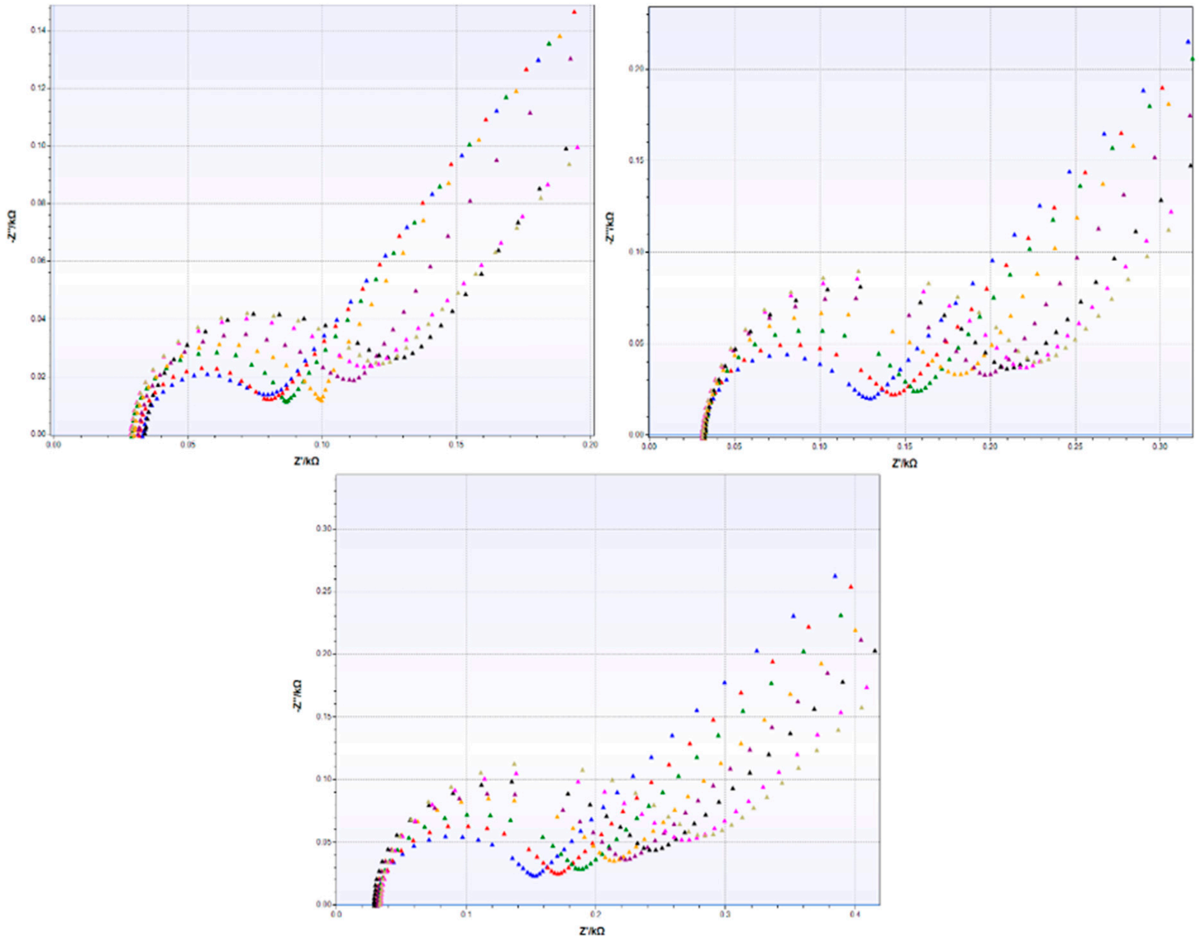

Figure S8. EIS spectra (Nyquist plots) obtained when testing CEA in PBS buffer using three CEA immunosensors. Each graph containing multiple EIS measurements represents the impedance signals of a single NSE immunosensor recorded for increasing concentrations of analyte. Legend: functionalized SPGE (Blue), 0 ng/ml (Red), 1 ng/ml (Green), 5 ng/ml (Yellow), 10 ng/ml (Purple), 20 ng/ml (Black), 50 ng/ml (Pink), 100 ng/ml (Khaki/Grey).

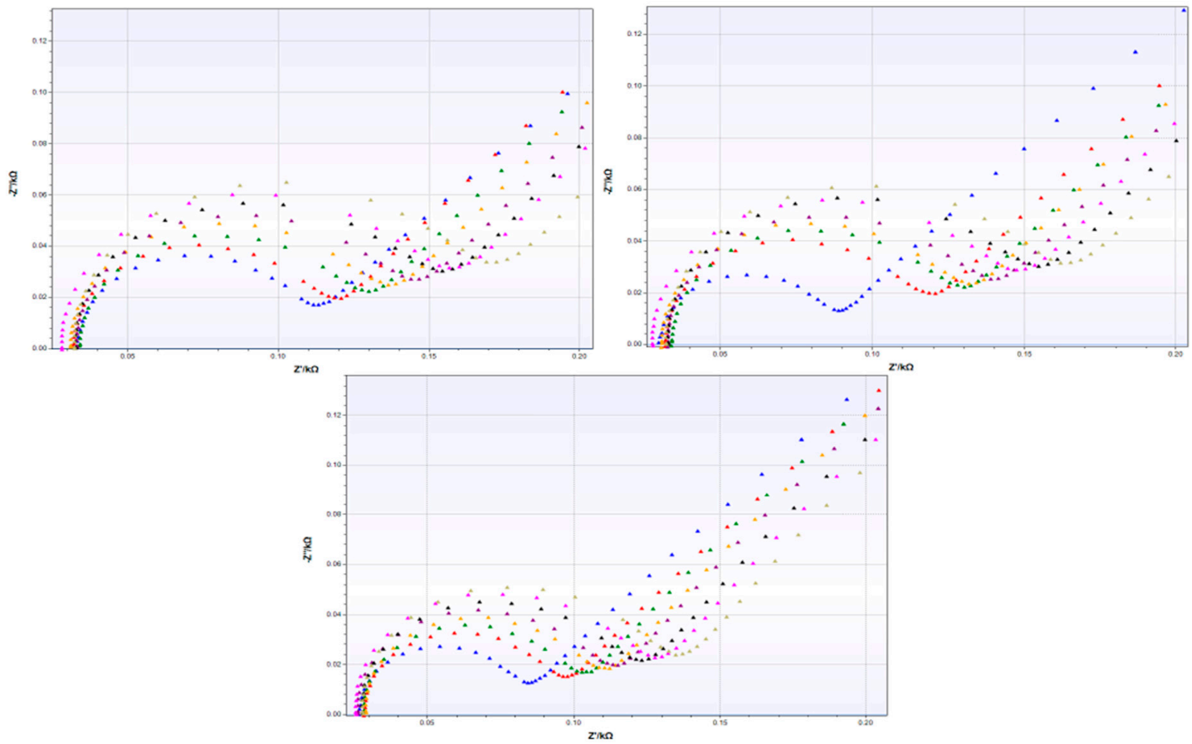

Figure S9. EIS spectra (Nyquist plots) obtained when testing NSE in PBS buffer using three NSE immunosensors. Each graph containing multiple EIS measurements represents the impedance signals of a single NSE immunosensor recorded for increasing concentrations of analyte. Legend: functionalized SPGE (Blue), 0 ng/ml (Red), 1 ng/ml (Green), 5 ng/ml (Yellow), 10 ng/ml (Purple), 20 ng/ml (Black), 50 ng/ml (Pink), 100 ng/ml (Khaki/Grey).

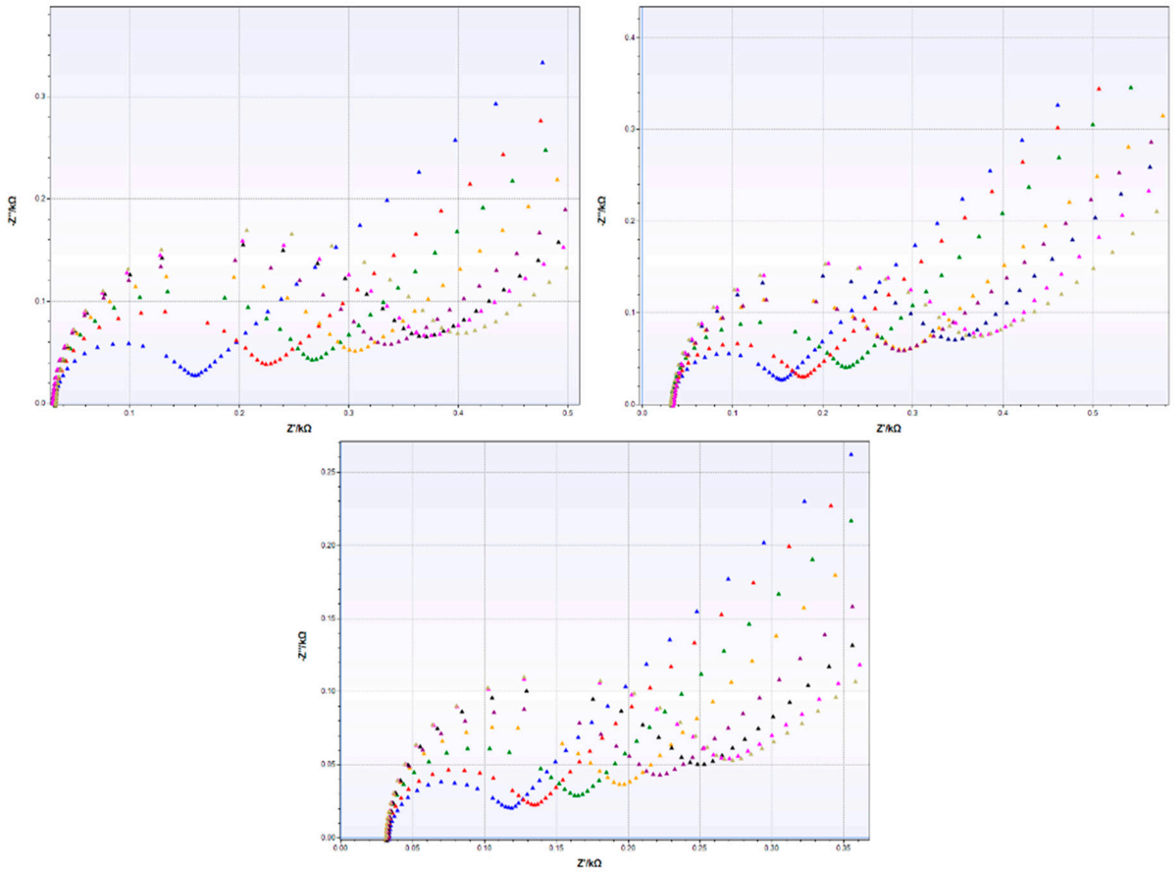

Figure S10. EIS spectra (Nyquist plots) obtained when testing CEA in serum using three CEA immunosensors. Each graph containing multiple EIS measurements represents the impedance signals of a single NSE immunosensor recorded for increasing concentrations of analyte. Legend: functionalized SPGE (Blue), 0 ng/ml (Red), 1 ng/ml (Green), 5 ng/ml (Yellow), 10 ng/ml (Purple), 20 ng/ml (Black), 50 ng/ml (Pink), 100 ng/ml (Grey/Khaki).

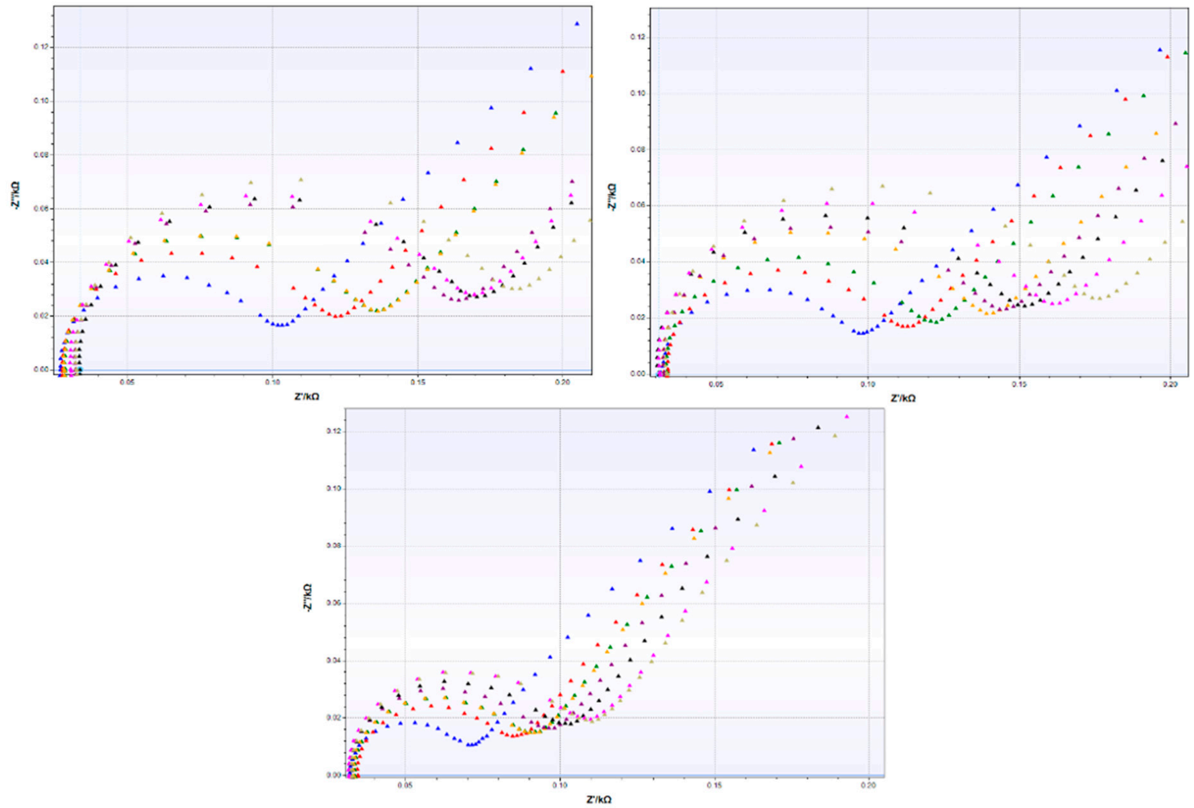

Figure S11. EIS spectra (Nyquist plots) obtained when testing NSE in serum using three NSE immunosensors. Each graph containing multiple EIS measurements represents the impedance signals of a single NSE immunosensor recorded for increasing concentrations of analyte. Legend: functionalized SPGE (Blue), 0 ng/ml (Red), 1 ng/ml (Green), 5 ng/ml (Yellow), 10 ng/ml (Purple), 20 ng/ml (Black), 50 ng/ml (Pink), 100 ng/ml (Khaki/Grey).

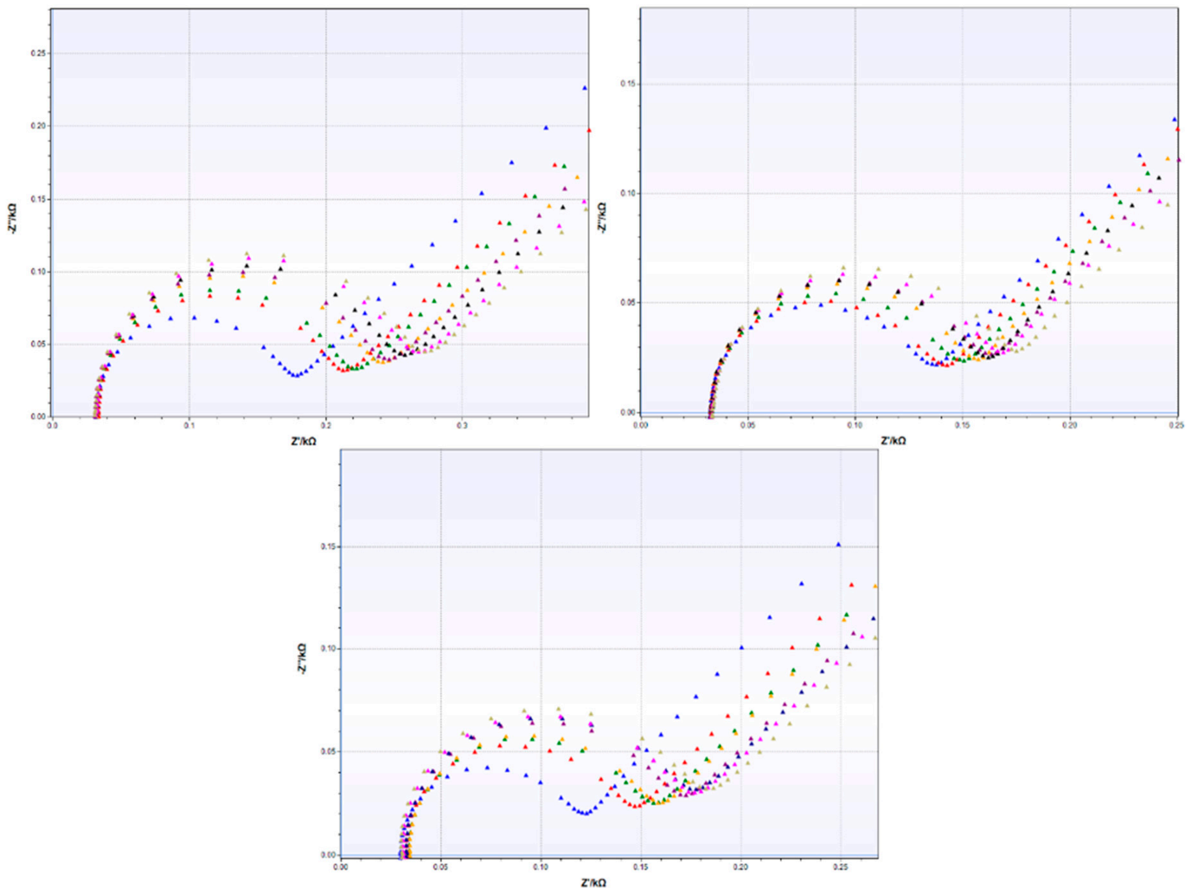

Figure S12. Cross-reactivity study - EIS spectra (Nyquist plots) obtained when testing NSE in serum using three CEA immunosensors. Each graph containing multiple EIS measurements represents the impedance signals of a single NSE immunosensor recorded for increasing concentrations of analyte. Legend: functionalized SPE (Blue), 0 ng/ml (Red), 1 ng/ml (Green), 5 ng/ml (Yellow), 10 ng/ml (Purple), 20 ng/ml (Black), 50 ng/ml (Pink), 100 ng/ml (Grey/Khaki).

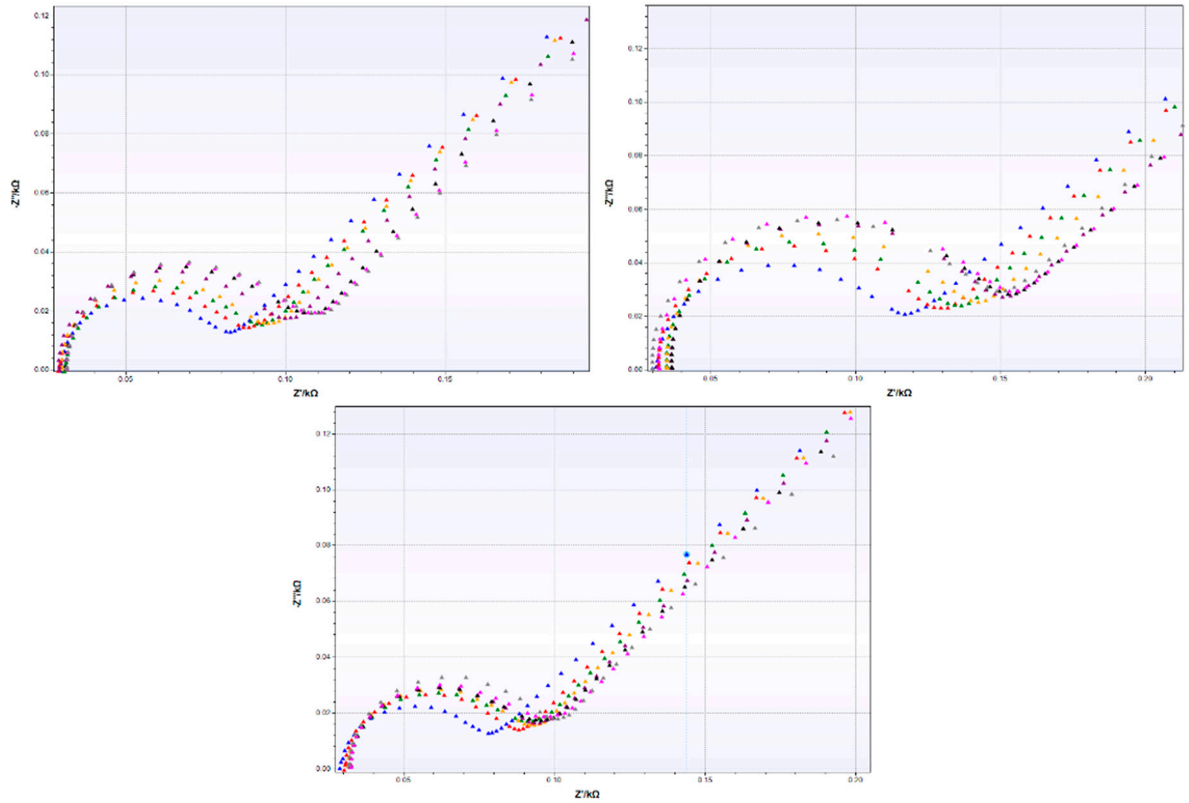

Figure S13. Cross-reactivity study - EIS spectra (Nyquist plots) obtained when testing CEA in serum using three NSE immunosensors. Each graph containing multiple EIS measurements represents the impedance signals of a single NSE immunosensor recorded for increasing concentrations of analyte. Legend: functionalized SPE (Blue), 0 ng/ml (Red), 1 ng/ml (Green), 5 ng/ml (Yellow), 10 ng/ml (Purple), 20 ng/ml (Black), 50 ng/ml (Pink), 100 ng/ml (Grey).
